# Use of the patientMpower App With Home-Based Spirometry to Monitor the Symptoms and Impact of Fibrotic Lung Conditions: Longitudinal Observational Study

**DOI:** 10.2196/16158

**Published:** 2020-11-20

**Authors:** Colin Edwards, Eamonn Costello, Nicola Cassidy, Bill Vick, Anne-Marie Russell

**Affiliations:** 1 patientMpower Ltd Dublin Ireland; 2 Irish Lung Fibrosis Association Dublin Ireland; 3 PF Warriors Plano, TX United States; 4 Imperial College Healthcare NHS Trust London United Kingdom; 5 University of Exeter College of Medicine and Health Exeter United Kingdom

**Keywords:** idiopathic pulmonary fibrosis, pulmonary fibrosis, eHealth, mHealth, patient-reported outcome measure, spirometry, home spirometry, patient experience, digital health, patient advocacy

## Abstract

**Background:**

Daily home-based spirometry in idiopathic pulmonary fibrosis (IPF) has been shown to be feasible and clinically informative. The patientMpower app facilitates home-based spirometry along with home-based monitoring of IPF-related symptoms. The patientMpower app can be downloaded to the user’s mobile phone or tablet device, enabling the recording of objective and subjective data.

**Objective:**

The aim of this paper is to report on the 1-year experience of using patientMpower with home-based spirometry by 36 participants with self-reported pulmonary fibrosis (PF) treated with usual care.

**Methods:**

Self-selecting participants enrolled in this community-based participatory research program through a patient advocacy group in their country: Irish Lung Fibrosis Association in Ireland and PF Warriors in the United States. Disease severity was comparable with a baseline mean predicted forced vital capacity (FVC) of 64% and 62% in the Irish and US participants, respectively. Both groups of participants were allocated to identical, in-country, open-label, single-group observational studies and were provided with a Bluetooth-active Spirobank Smart spirometer integrated directly with patientMpower. Data collected via patientMpower included seated FVC (daily), breathlessness grade (modified Medical Research Council scale score), step count, medication adherence, and symptoms and impact of IPF on daily life, which were measured by a patient-reported outcome measure (PROM) scale that was specifically developed for IPF. Longitudinal patient-reported data on oximetry and oxygen consumption were also collected.

**Results:**

A large majority of the 36 participants reported that their experience using patientMpower was positive, and they wanted to continue its use after the initial 6-week observation. Out of 36 participants, 21 (58%) recorded home-based spirometry without prompting for ≥180 days, and 9 (25%) participants continued with recording home-based spirometry for ≥360 days.

**Conclusions:**

The patientMpower app with associated Bluetooth-connected devices (eg, spirometer and pulse oximeter) offers an acceptable and accessible approach to collecting patient-reported objective and subjective data in fibrotic lung conditions.

## Introduction

Pulmonary fibrosis (PF) is an irreversible lung disease that leads to progressive breathlessness and deterioration of pulmonary function; it is associated with significant limitation of physical activities and worsening quality of life [1]. The majority of PF cases are idiopathic, and there is no cure. The advent of disease-modifying/antifibrotic pharmacotherapies in the last decade has slowed the progression of PF, with registry data reporting an increase in median survival from 3 to 4.5 years. The progression of PF is highly variable. Acute exacerbations resulting in further impairment of lung function are a major cause of morbidity and mortality in idiopathic pulmonary fibrosis (IPF), reducing median survival to <21 months where hospitalization is needed [2].

Adjunctive symptom-based management is integral to disease management given the high symptom burden [3]. Objective assessments of lung function and symptom experience (including breathlessness and fatigue) are important in the monitoring of patients with IPF to inform both their self-management and hospital-based care plan, particularly initiation and adjustment of oxygen therapy, pulmonary rehabilitation, palliative care, and psychological therapies [4]. Traditional monitoring of IPF has typically involved spirometric measurement of forced vital capacity (FVC) along with the assessment of IPF-related symptoms at 6- and 12-month intervals in outpatient clinical settings.

Digital platforms which directly record patients’ symptom experiences and impacts of those symptoms on patients’ daily lives alongside objective clinical measurements (eg, blood pressure) have been developed for chronic medical conditions to support patients in managing their health. There was a lack of digital platforms created specifically for patients with IPF, and there were few disease-specific instruments to assess health-related quality of life with IPF. The IPF patient-reported outcome measure (IPF-PROM) captures symptom experience and impact of IPF on patients’ daily lives [5]. The collection of serial IPF-PROM data provides valuable information on longitudinal trends at individual and population levels. Furthermore, home-based monitoring of FVC by patients using small handheld spirometers is feasible, clinically informative, and more accurate in estimating lung function in patients with IPF than intermittent spirometry performed at clinic visits, and it may be of value as a primary endpoint in short proof-of-concept studies in IPF [6-8].

The patientMpower app was developed to facilitate home-based spirometry and symptom monitoring in PF. Users download patientMpower to their mobile phones or tablet devices, enabling them to record both objective data (eg, FVC) and subjective data (eg, impact on quality of life) simultaneously. There are few published data on longitudinal trends in patient-measured FVC and patient-reported outcome measures (PROMs) in IPF. Patient-reported data may predict important health outcomes (eg, exacerbations). We have previously reported on 6-week feasibility studies of patientMpower in patients with PF [9,10] These studies were codesigned in collaboration with patient advocacy groups (in Europe and the United States). Here, we present the 1-year follow-up of these studies designed to test the acceptability and utility of patientMpower.

## Methods

We conducted 2 parallel studies of the same design and methodology in 2 distinct populations (in the United States and Ireland). The studies were codesigned and conducted in collaboration with 2 advocacy groups for patients with PF: PF Warriors, Texas, United States, and Irish Lung Fibrosis Association (ILFA), Dublin, Ireland. Our approach was not prescriptive, as we aimed to observe the real-world experience of the integration of patientMpower into the daily lives of people with fibrotic lung conditions.

The patient advocacy groups collaborated with us from the outset of this study, informing, reviewing, and approving the study concept and design and leading on the patient information documents prior to the initiation of the study. The study protocol evolved iteratively, informed by the stakeholder and participant discussions and by peer review by health care professionals. Our approach was not that of conventional science and was rather founded in an implementation science approach, working “with” participants instead of “on” them. Participatory action research seeks to empower participants to tailor an intervention to suit their own contexts [11]. We were keen to ensure that patientMpower fitted into the context of an individual’s life. When using such approaches, the area between engagement/consultation and research is gray. Given that this work was led by patient advocacy groups and was focused on increasing experiential knowledge and obtaining results within a timeframe to determine how to scale up the use and sustainability of patientMpower, we were informally advised that approval from an institutional review board was not required. Nonetheless, in line with principles of good clinical practice, all participants provided written informed consent prior to accessing patientMpower and participating in any study-specific procedures. Entry criteria included a diagnosis of PF, ownership of a smartphone or tablet device, an email address, access to the internet at home, and willingness to provide written informed consent.

Each study was an open-label, single group, observational study in patients with PF treated with usual care. After obtaining consent, participants were invited to download the patientMpower app and use it daily for ≥6 weeks (the active observational study period). All participants were provided with a Spirobank Smart spirometer (Medical International Research), which integrates directly via Bluetooth with patientMpower to capture spirometry data. FVC data were displayed to each participant on their mobile device. Video-based training on the correct use of patientMpower and home-based spirometry was provided on a dedicated YouTube channel [12]. Participants were asked to record the following data via the app: FVC (one forced expiratory maneuver per day, seated), breathlessness (using the modified Medical Research Council [mMRC] score, once a day), step count, medication adherence, symptoms, and impact of IPF on daily life (using PROM scores) [5]. The IPF-PROM consisted of an overall quality of life question and 12 questions covering 4 domains: physical impacts of breathlessness, psychological impacts of breathlessness, psychological well-being, and fatigue. There were no prompts to record spirometry or breathlessness; however, participants were prompted to record a PROM once per week for the first 6 weeks.

Participants in the United States could record pulse oximetry on patientMpower using a Nonin 3230 Bluetooth-linked pulse oximeter (Nonin Medical Inc) and oxygen flow rates. The protocol did not specify the frequency or conditions for recording pulse oximetry, and the pulse oximeter was not supplied by the study sponsor.

At the end of the initial 6-week observation, participants were asked to complete a questionnaire to assess the utility and acceptability of patientMpower (Multimedia Appendix 1). Thereafter, participants were free to use patientMpower and the spirometer if they chose.

The sample sizes for each study were determined by estimating the number of participants that could be recruited by the advocacy groups over a 2-month period. We expected 50 participants to be enrolled by the PF Warriors and 30 participants from the ILFA.

The results are tabulated and discussed in the following section. Anonymized demographic data are displayed for all participants who gave informed consent. All other displayed data are for participants who used patientMpower and recorded spirometry at least once.

The data sets used or analyzed in this study are available from the corresponding author on reasonable request.

## Results

### Study Population

In the United States, 27 participants enrolled in the study; 24 used patientMpower and recorded spirometry at least once. In Ireland, 13 participants enrolled in the study, all used patientMpower, and 12 recorded spirometry at least once. Baseline demographics were comparable in both groups (Table 1). Most US study participants (25/27, 93%) reported a confirmed diagnosis of PF; this information was not reported by Irish study participants. Most US participants (23/27, 85%) and some Irish participants (4/13, 31%) reported use of antifibrotic therapy at baseline.

**Table 1:** Baseline demographic data.

Characteristics		US study participants (N=27)	Ireland study participants (N=13)
**Gender, n (%)**			
	Male	12 (44)	7 (54)
	Female	13 (48)	6 (46)
	Not stated	2 (7)	0 (0)
**Ethnicity, n (%)**			
	White	24 (89)	13 (100)
	Other	1 (4)	0 (0)
	Not stated	2 (7)	0 (0)
Age (years), mean (range)		62 (31-79)	66 (37-81)
FVC^a,b^ (L), mean (range)		2.28^c^ (0.6-4.72)	2.49^d^ (0.7- 3.9)
FVC^b^ (% predicted), mean (range)		62 (36-108)	64 (33-91)
**Diagnosis confirmed by clinical expert^e^, n (%)**			
	Yes	25 (93)	0 (0)
	No	0 (0)	0 (0)
	Not stated	2 (7)	13 (100)
**On antifibrotic therapy^e^, n (%)**			
	Yes	23 (85)	4 (31)
	No	0 (0)	0 (0)
	Not stated	4 (15)	9 (61)

^a^FVC: forced vital capacity.

^b^Home-based spirometry data.

^c^Mean of first 7 days in 23 US participants. A total of 24 US participants provided home-based spirometry data; however, 1 participant did not provide spirometry data in the first 7 days.

^d^Mean of first 7 days in 12 Irish participants who provided home-based spirometry data.

^e^Self-reported data.

### User-Reported Experience at 6 Weeks

At 6 weeks, 75% (18/24) of the US participants and 92% (11/12) of the Irish participants completed a questionnaire. Analysis of the 29 completed responses indicated that a large majority of respondents liked using patientMpower (28/29, 96%) and found it easy to use (26/29, 90%). A majority reported that patientMpower helped them take the correct dose of medication at the prescribed time and achieve personal exercise goals (19/29, 66%). Most reported that the effect of using patientMpower on their well-being and daily life was positive (27/29, 93%) and that it was useful to be able to record the impact of lung fibrosis on their well-being and daily life (25/29, 86%). Most participants stated they would recommend patientMpower to others and wanted to continue using it after the initial 6 weeks (27/29, 93%).

### Frequency of Home-Based Spirometry Recording

**Figure 1:**
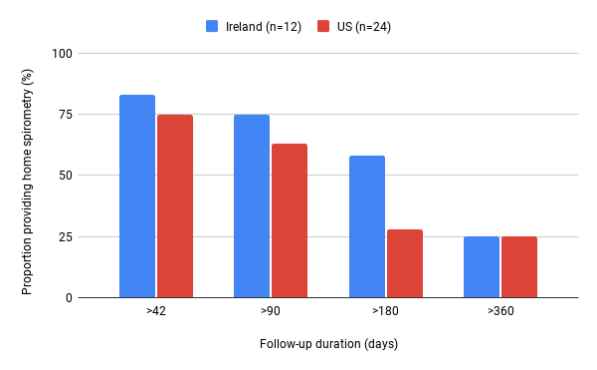
The proportion of participants from the Ireland and US studies recording home-based spirometry at least once against the duration of follow-up. Proportion of participants providing home-based spirometry data at each follow-up duration calculated relative to total numbers of participants who provided any spirometry data in that population. US: United States.

A total of 24 US participants and 12 Irish participants recorded home-based spirometry, and most (28/36, 78%) continued home-based spirometry after the initial 6-week observation (Figure 1). Out of 36 participants, 21 (58%) recorded home-based spirometry at least once after 180 days, and 9 (25%) recorded it at least once after 360 days. There was variation in the frequency and duration of recording of home-based spirometry, with some participants recording spirometry daily and others recording intermittently but over a long duration of follow-up (data not shown). A total of 39% (14/36) of participants recorded home-based spirometry at least once a week on ≥50% of weeks over 1 year.

### Recording Health Outcomes

A total of 22 US and 13 Irish participants completed at least 1 IPF-PROM. There was a trend toward a greater number of IPF-PROMs reported by US participants compared with Irish participants (mean 12/person vs 7/person, respectively). More US participants (13/22, 59%) provided IPF-PROMs after 180 days compared to Irish participants (3/13, 23%).

Breathlessness data (using mMRC score) were provided by 15 US participants and 11 Irish participants. Most participants reported breathlessness at baseline (median mMRC score for Irish participants=1; US participants=3) with only a few (2/11 in the Irish study and 2/15 in the US study) reporting an mMRC score of 0 (ie, dyspnea only with strenuous exercise). A greater number of breathlessness data reports were recorded by US participants compared to Irish participants (mean 28/person in the US study vs 12/person in the Ireland study). A similar proportion of participants in the Irish and US study populations recorded a breathlessness score at least once after 180 days (6/15 US participants, 40%, vs 5/11 Irish participants, 45%). There were no reports of exacerbation of IPF or death in the study populations.

### Pulse Oximetry

Pulse oximetry data were not available for the Irish population, as Bluetooth integration of the pulse oximeter with patientMpower had not been implemented at that time. In the US study, 20 participants recorded pulse oximetry. The mean number of recordings was 58/person over a duration ranging from 1 to 409 days. A total of 10 participants recorded oximetry for ≥180 days.

**Figure 2:**
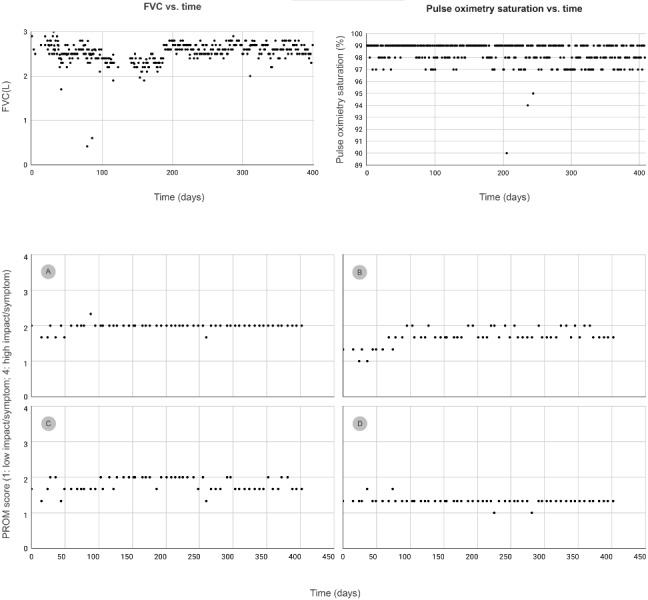
Patient-recorded spirometry, oximetry, and PROM in a single participant over 1 year in 4 domains (A) psychological impact of breathlessness, (B) psychological well-being, (C) physical impact of breathlessness, and (D) fatigue. FVC: forced vital capacity; PROM: patient-reported outcome measure.

Figure 2 shows an example of multiple data recorded by a single US participant over approximately 400 days. This participant's self-recorded baseline FVC was 65% predicted, and they reported antifibrotic therapy (nintedanib 150 mg twice daily). This participant reported breathlessness (mMRC score 1 or 2) with a regular use of oxygen (flow rate 3 or 4 L/min) throughout. They described their overall quality of life in the PROM as “good” throughout the follow-up.

Figure 2 displays all patient-recorded values of FVC and pulse oximetry with no exclusion of outliers. Data on breathlessness (using mMRC scores) and oxygen flow rates were also collected (however, these are not shown in Figure 2). There were 4 grades of severity of a symptom or an impact of a symptom on quality of life for each question in the PROM. A score of 1 indicates lowest severity or impact (ie, the patient experienced the symptom/impact “none of the time” in the previous week). A score of 2 implies that the symptom/impact was experienced “some of the time,” and a score of 3 implies that the symptom/impact was experienced “most of the time.” A score of 4 indicates highest severity or impact (ie, the symptom/impact was experienced “all of the time” in the previous week). The scores for the 3 questions were averaged for each domain (psychological impact of breathlessness in panel A, psychological well-being in panel B, physical impact of breathlessness in panel C, and fatigue in panel D).

## Discussion

Patients with chronic conditions (including PF) may be interested in using electronic tools to help them monitor and manage their health. The results we observed are consistent with those reported in a study in the Netherlands [13] in which a sample of patients with IPF were asked if they would welcome a web-based platform to track their health; 67 (82%) responded “yes.” The Dutch web-based platform was evaluated in a study [14] with 27 patients with IPF who recorded medication use and PROM at baseline and after 14 days. A large majority of these patients were “very satisfied” with their experience of this platform. This research team [14] went on to develop a home-based monitoring program using the MIR Spirobank Smart spirometer (the same device used in our studies); however, the FVC data were displayed on the patient’s personal computer rather than on their mobile device. The FVC data were evaluated in 10 patients with IPF who recorded daily home-based spirometry and symptoms for 4 weeks. Adherence over 4 weeks was very high (99%), with good correlation between home-based and clinic spirometry records. All patients reported that home-based spirometry was useful for them; we replicate these findings here in larger numbers.

Patient-reported spirometry in patients with IPF has been evaluated and found to be feasible in other studies and, in general, it showed good correlation with clinic-measured spirometry [6,7]. In one study [6], correlation was observed between clinic spirometry at 3, 6, and 12 months, and the rate of decline of patient-recorded FVC was predictive of clinical outcome.

In another study, weekly home-based spirometry was evaluated in 25 patients with IPF using the best of 3 forced expiratory maneuvers with data captured directly by the spirometer (rather than in a paper diary) [7]. Mean adherence to weekly spirometry was 90.5% over the 24-week observation period, decreasing thereafter. The study [7] also reported good correlation between patient-measured and clinic spirometry data. The authors hypothesized that use of home-based patient-recorded spirometry data could improve the analytical efficiency of clinical trials and reduce the sample size required (vs clinic spirometry). This has proven to be of particular importance in the era of COVID-19.

The patientMpower app with integrated home-based spirometry has been used in clinical studies in patients with interstitial lung disease. In the first pilot-scale clinical study (n=7) of patientMpower for patients with IPF (recruited through a specialist interstitial lung disease clinic), there was a good correlation between home-based and clinical FVC recordings at 8 weeks [15]. At 8 months after the conclusion of that study, 3 of the 6 participants were still using patientMpower regularly. Other clinical studies in populations with interstitial lung disease reported good adherence to use of the patientMpower app with home-based spirometry [16,17].

There are several limitations to our study. The sample size was chosen arbitrarily (based on estimates of expected recruitment), and actual recruitment was lower than anticipated. Participants self-reported their diagnosis of PF, medication history, and other demographic data without clinical confirmation of these data. The participants were recruited through patient advocacy groups and were likely to represent highly engaged patients with a strong interest in monitoring and managing their health. The level of engagement demonstrated by the participants may not be typical of the general population of patients with PF. The questionnaire used to assess participants’ opinions was developed de novo by the study sponsor and has not been tested in other populations.

A possible future research use of patientMpower would be the assessment of long-term patient-recorded spirometry and quality of life to better understand the correlation between lung function and subjective outcomes in chronic respiratory conditions. Inclusion of occasional formal clinic assessment of lung function and exercise capability (eg, 6-minute walking distance) would enhance the interpretation of study data (although in-clinic assessments are now much more difficult to access during the COVID-19 pandemic). The patientMpower app can be adapted so that clinical centers can view their patients’ recorded data in real time to create a remote patient-monitoring approach. The patientMpower app with remote monitoring of pulse oximetry has already been implemented in lung transplantation centers and in a national program to support early discharge of patients diagnosed with COVID-19 from in-hospital care [18].

The age profiles of the patients in all of these studies were typical of IPF, implying that age per se is not a barrier to the use of home-based spirometry or electronic platforms to track health outcomes. To the best of our knowledge, our studies [9,10,15,17] are the first to report use of a specific mobile app by patients with IPF to capture and view their health data directly within a single platform. Patients with IPF appear to be motivated to record home-based spirometry regularly, even when not prompted.

Recruiting volunteers through patient support groups to participate in observational studies is feasible. Our work has shown that electronic informed consent and remote installation of health care apps (with associated sensor devices) can be implemented in observational studies for some patient populations with PF. This approach may be useful to capture patient-reported long-term trends in FVC, quality of life, and health outcomes in patients with PF and generate richer real-world evidence. This is illustrated by Figure 2 where the participant recorded objective data (FVC and oximetry) and subjective data (breathlessness and impact of IPF on quality of life) simultaneously at multiple time points over 1 year. The patientMpower app can also capture data on step count (from the user’s smartphone or a connected FitBit device), enabling future analysis of activity levels in addition to the reported outcomes. To the best of our knowledge, presently, patientMpower is the only mobile app designed specifically for patients with PF that enables capture of all PF-relevant health outcomes within a single app.

Long-term use of the patientMpower app with integrated Spirobank Smart spirometry to record daily FVC, symptoms, and other health outcomes is acceptable and feasible for patients with PF. Patients with PF show willingness to record home-based spirometry data regularly without prompting, which suggests that they are interested in monitoring their lung function. Some are willing to continue recording home-based spirometry and other outcomes on a long-term basis even when not involved in a formal clinical trial or survey. When prompts to record other health outcomes (eg, PROM) are added, these prompts have resulted in regular reporting of those outcomes. It is anticipated that use of prompts or triggers to record spirometry will result in more sustained and frequent collection of home-based spirometry data.

This study was led by patient advocacy groups, and all participants used the patientMpower app independently without help from health care professionals. Our observations suggest that using a mobile device-based app linked to appropriate sensor devices is feasible to capture multiple long-term patient-reported objective and subjective outcomes within a single platform for studies in pulmonary fibrosis. This work was undertaken well ahead of the COVID-19 pandemic announced in March 2020. Our findings have informed the longitudinal clinical studies on the home-based monitoring of patients with fibrotic lung diseases to guide medication adjustments and nonpharmacological support strategies.

## Appendix

Multimedia Appendix 1 Description of the participant questionnaire.

## Abbreviations

FVC: forced vital capacity
ILFA: Irish Lung Fibrosis Association
IPF: idiopathic pulmonary fibrosis
IPF-PROM: IPF patient-reported outcome measure
mMRC: modified Medical Research Council
PF: pulmonary fibrosis
PROM: patient-reported outcome measure
