# Supplementary material for: Use of the patientMpower App With Home-Based Spirometry to Monitor the Symptoms and Impact of Fibrotic Lung Conditions: Longitudinal Observational Study
Source: JMIR Mhealth Uhealth. 2020 Nov 20;8(11):e16158. doi: 10.2196/16158 (PMC7718088; doi:10.2196/16158)
Supplement: Multimedia Appendix 1 [file mhealth_v8i11e16158_app1.doc]

**Supplementary material MS#16158**

**Title:**

#### **The utility of an electronic health journal with home spirometry in a real world population of people with fibrotic lung conditions.**

**Description of questionnaire to assess utility and acceptability of the patientMpower application:**

At the end of the initial six-week observation period, patients were asked to complete the following questionnaire:

1. the instructions given in using the patientMpower platform were clear and easy to understand (strongly agree/agree/disagree/strongly disagree)
2. using the patientMpower platform helped me to take the correct dose of my medicines every day (strongly agree/agree/disagree/strongly disagree)
3. using the patientMpower platform helped me to take my medicines at the correct time every day (strongly agree/agree/disagree/strongly disagree)
4. using the patientMpower platform motivated me to reach my personal exercise goal every day (strongly agree/agree/disagree/strongly disagree)
5. using the patientMpower platform helped me to walk further (or exercise more) compared with before (strongly agree/agree/disagree/strongly disagree)
6. using the patientMpower platform gave me more confidence/a greater sense of control in managing my lung health (strongly agree/agree/disagree/strongly disagree)
7. I found it useful to be able to record the impact of lung fibrosis on my well-being and daily life (strongly agree/agree/disagree/strongly disagree)
8. using the patientMpower platform encouraged me to look at the information videos on the platform (strongly agree/agree/disagree/strongly disagree)
9. I liked using the patientMpower platform (strongly agree/agree/disagree/strongly disagree)
10. the patientMpower platform was easy to use (strongly agree/agree/disagree/strongly disagree)
11. the effect of using the patientMpower platform on the impact of lung fibrosis on my well-being and daily life was (positive, negative, optional open text field for participant to give opinion)
12. I found it tiring or irritating to use the patientMpower platform (strongly agree/agree/disagree/strongly disagree)
13. I want to continue using the patientMpower platform after the end of the study (yes/no)
14. I would recommend other people with my condition to use the patientMpower platform (yes/no)
15. what other measurements, reminders or information would be useful to have on the patientMpower platform? (optional open text field for participant to give opinion)
16. describe the benefits and/or disadvantages of using the patientMpower platform (optional open text field for participant to give opinion)
17. any other comments on the patientMpower platform (optional open text field for participant to give opinion)

Respondents could provide only one possible response to questions 1-10, 12-14.

Respondents could provide a one possible response (positive or negative) and add free text to question 11.

Respondents could add (or not) free text in response to questions 15-17.
